# A Heartbeat Away From Consciousness: Heart Rate Variability Entropy Can Discriminate Disorders of Consciousness and Is Correlated With Resting-State fMRI Brain Connectivity of the Central Autonomic Network

**DOI:** 10.3389/fneur.2018.00769

**Published:** 2018-09-12

**Authors:** Francesco Riganello, Stephen Karl Larroque, Mohamed Ali Bahri, Lizette Heine, Charlotte Martial, Manon Carrière, Vanessa Charland-Verville, Charlène Aubinet, Audrey Vanhaudenhuyse, Camille Chatelle, Steven Laureys, Carol Di Perri

**Affiliations:** ^1^Coma Science Group, GIGA-Consciousness, University & Hospital of Liege, Liege, Belgium; ^2^Research in Advanced NeuroRehabilitation, Istituto S. Anna, Crotone, Italy; ^3^GIGA-Cyclotron Research Center in vivo Imaging, University of Liege, Liege, Belgium; ^4^Centre de Recherche en Neurosciences, Inserm U1028 - CNRS UMR5292, University of Lyon 1, Bron, France; ^5^Sensation & Perception Research Group, GIGA-Consciousness, University & Hospital of Liege, Liege, Belgium; ^6^Centre for Clinical Brain Sciences, University of Edinburgh, Edinburgh, United Kingdom

**Keywords:** heart rate variability entropy (HRV), disorders of consciousness (DOC), unresponsive wakefulness syndrome/vegetative state (UWS/VS), minimally conscious state, functional connectivity, resting-state fMRI, machine learning

## Abstract

**Background:** Disorders of consciousness are challenging to diagnose, with inconsistent behavioral responses, motor and cognitive disabilities, leading to approximately 40% misdiagnoses. Heart rate variability (HRV) reflects the complexity of the heart-brain two-way dynamic interactions. HRV entropy analysis quantifies the unpredictability and complexity of the heart rate beats intervals. We here investigate the complexity index (CI), a score of HRV complexity by aggregating the non-linear multi-scale entropies over a range of time scales, and its discriminative power in chronic patients with unresponsive wakefulness syndrome (UWS) and minimally conscious state (MCS), and its relation to brain functional connectivity.

**Methods:** We investigated the CI in short (CI_s_) and long (CI_l_) time scales in 14 UWS and 16 MCS sedated. CI for MCS and UWS groups were compared using a Mann-Whitney exact test. Spearman's correlation tests were conducted between the Coma Recovery Scale-revised (CRS-R) and both CI. Discriminative power of both CI was assessed with One-R machine learning model. Correlation between CI and brain connectivity (detected with functional magnetic resonance imagery using seed-based and hypothesis-free intrinsic connectivity) was investigated using a linear regression in a subgroup of 10 UWS and 11 MCS patients with sufficient image quality.

**Results:** Higher CI_s_ and CI_l_ values were observed in MCS compared to UWS. Positive correlations were found between CRS-R and both CI. The One-R classifier selected CI_l_ as the best discriminator between UWS and MCS with 90% accuracy, 7% false positive and 13% false negative rates after a 10-fold cross-validation test. Positive correlations were observed between both CI and the recovery of functional connectivity of brain areas belonging to the central autonomic networks (CAN).

**Conclusion:** CI of MCS compared to UWS patients has high discriminative power and low false negative rate at one third of the estimated human assessors' misdiagnosis, providing an easy, inexpensive and non-invasive diagnostic tool. CI reflects functional connectivity changes in the CAN, suggesting that CI can provide an indirect way to screen and monitor connectivity changes in this neural system. Future studies should assess the extent of CI's predictive power in a larger cohort of patients and prognostic power in acute patients.

## Introduction

Disorders of consciousness are a spectrum of pathologies affecting one's ability to interact with the external world. They are increasingly becoming a worldwide health concern, whether of traumatic (1, 2) or non-traumatic (3–6) cause, with its share of ethically challenging questions including life and death decisions (7–9). Indeed, differential diagnosis of the clinical entities of disorders of consciousness raises crucial ethical and medical issues, including pain treatment and end-of-life decisions (8, 10, 11).

Despite the definition of such a unified name, these disorders are in fact covering a broad population of very heterogeneous pathologies with diverse etiologies, injuries and outcomes. This heterogeneity can make them hardly distinguishable in the clinical practice (9), leading to a reported misdiagnosis rate between 33 and 41% for the clinical consensus (12, 13). Although the clinical characterization of disorders of consciousness can now be more reliably assessed using specifically designed scales such as the Coma Recovery Scale-Revised (CRS-R) (14), practicing them requires a specific training of the physicians and, although lower, might still induce diagnosis errors inherent to any behavior-based clinical assessment due to the patient's possible inability to respond (13). Indeed, these assessments rely on observing the patient's motor actions, and their absence does not necessarily relate to the absence of consciousness, as there are several other factors that might hamper the patient's responsiveness to the assessment (motor disabilities, language understanding difficulties, fluctuating consciousness because of natural awareness fluctuations or the influence of drugs side effects, patient's willingness to collaborate among other factors) (13). Neuroimaging has been proposed as a complementary tool to help in assessment and decision making for these critical conditions (13, 15, 16). However, these techniques are usually highly costly, complex, and time consuming. Alternative methods, such as probing physiological signals of peripheral organs like the heart, have been proposed to overcome these issues (17–19).

Heart rate is defined as the numbers of heartbeats per minute; the Heart Rate Variability (HRV) is the fluctuation in the time intervals between adjacent heartbeats. These fluctuations represent the output of a complex brain-heart two-way interaction system (20–22). Indeed, HRV analysis provides a window into the brain's function. HRV has been observed to rapidly and flexibly modulate response to environmental changes and can be disrupted by neurological and non-neurological diseases usually involving the autonomic nervous system (23–29). The HRV recording technique is non-invasive, inexpensive to acquire and has an excellent signal-to-noise ratio compared to signals investigated in neuroimaging or clinical neurophysiology (30).

HRV is analyzed in time and frequency domains and by non-linear methods (31). In the time domain, this is quantified by the amount of heartbeats variability observed during monitoring periods in the range of 1 min to more than 24 h. In the frequency domain, HRV is calculated as the absolute or relative amount of signal energy within the component bands. Fast Fourier Transformation (FFT), Auto-regression or Wavelet modeling are used to separate the HRV into its main components: Ultra Low Frequency (ULF), Very Low Frequency (VLF), Low Frequency (LF), and High Frequency (HF) (31).

As the sequence of heart beats is not regular and exhibit complex fluctuation patterns over a wide range of time scales, HRV is better described by the mathematical chaos (32, 33), therefore non-linear analyses are appropriate to model this type of time series. These analyses quantify the unpredictability and complexity of the interbeat intervals (IBI) series. Poincare plot (34), detrended fluctuation analysis (35), approximate entropy (36), sample entropy (SE) (37), and multiscale entropy (MSE) (38) are among the most commonly applied methods of non-linear analysis used in the HRV analysis.

MSE was developed to investigate the information content in non-linear signals at different temporal scales (coarse-graining), using generally the SE in order to quantify the degree of unpredictability of time series. In other words, applying MSE on top of the HRV allows to measure the diversity of the heart beat intervals: higher entropy indicates a more unpredictable and diverse heart beats sequence, and conversely lower entropy indicates a more regular and predictable heart beats. Considering the complex brain-heart interactions system mentioned above, it is conceivable that the HRV entropy might be a way to measure the health status of this system, with a low value being indicative of low reactivity to the external/internal stimulus. Indeed, MSE on HRV was shown to be a marker of health status of biological systems (39–41). The Complexity Index (CI) is calculated from the MSE measures and is defined as the sum of the entropies computed for different scales (i.e., at different levels of resolution of the signal). The CI thus provides a scalar score, which is the aggregation of MSE over multiple time scales, and it allows to get insights into the integrated complexity of the measured system (41).

Heart rate, as well as respiration rate, glands, smooth muscles functions and biological sensors are under the control of the Autonomic Nervous System (ANS), which is in charge of maintaining the homeostasis without any conscious control (42). The sympathetic (“fight or flight system”) and parasympathetic (“rest and digest” system) branches of the ANS have an antagonistic role and are connected to the brain by the spinal nerves (43). By doing so, they modulate the ANS functional status through inputs from thermoregulation, baroreceptors, chemoreceptors, renin-angiotensin-aldosterone balance and atrial and ventricular receptors (18, 44–46).

The Central Autonomic Network (CAN) has been proposed as an integrative model where neural structures and heart function are involved and functionally linked in the affective, cognitive and autonomic regulation (47, 48). The CAN is defined as covering the structures of the brainstem (periaqueductal gray matter, nucleus ambiguous, and ventromedial medulla), limbic structure (amygdala and hypothalamus), prefrontal cortex (anterior cingulate, insula, orbitofrontal, and ventromedial cortex) and cerebellum (22, 49, 50). Some brain regions of the CAN (dorsolateral prefrontal cortex, mediodorsal thalamus, hippocampus, caudate, septal nucleus and middle Temporal Gyrus) seem to be unique to humans (51–53). The interplay between Central Nervous System (CNS) and ANS is functionally modeled as a setup involving the above-cited structures connecting to the brainstem solitary tract (NTS) via feed-forward and feedback loops. These coupled structures and their oscillatory signals, integrated in the NTS by the efferent parts of the vagus nerve, are coupled with organs outside the brain in a bidirectional way. Through this two-way interaction, peripheral oscillations, such as those in the heart, lung, immunological system and kidney, can lead to changes in the CAN, as well as be influenced by the CAN (54–57). HRV measurements are thought to reflect heart rate interaction and ANS dynamics and, to some extent and indirectly, higher brain functions (58–61), and thus might be relevant for diagnostic purposes (62, 63).

In the present study, we aimed to characterize and investigate the discriminative power of the CI in sedated patients suffering from disorders of consciousness, more specifically diagnosed as either unresponsive wakefulness syndrome (UWS, i.e., vegetative state—eye opening without signs of awareness) or minimally conscious (MCS—displaying non-reflexive behaviors) according to the CRS-R clinical assessment. In the light of the above mentioned studies, we hypothesized an impaired two-way brain heart connection (due to the loss of the biological complexity linked to physiologic mechanism) (14, 58), and consequently lower values of CI in UWS patients on average compared to MCS. We further expected CI values to be correlated with each patient's behavioral assessment as measured with the Coma Recovery Scale Revised (CRS-R) (14). In addition, we expected the CI measures to possess some discriminative power on the diagnosis when used in a machine learning model such as One-R classifier, an algorithm deriving a single association rule between the most discriminating feature and the diagnosis classification (64).

With the aim of investigating brain regions' involvement in the HRV entropy, we further investigated the relationship between the CI measures and the brain connectivity patterns, and whether there are different patterns for UWS and MCS that are correlated with changes in the CI values. In this optic, we correlated, using a linear parametric regression, the per-subject CI values with brain regions connectivity patterns as detected by whole-brain resting-state functional magnetic resonance imagery (fMRI). fMRI is a non-invasive technique used to investigate the spontaneous temporal coherence in blood-oxygen-level dependent (BOLD) signal fluctuations related to the amount of synchronized neural activity (i.e., functional connectivity) existing between distinct brain locations (65). Combined with a regression of the physiological noise by principal components analysis via aCompCor, this approach, novel in its application to HRV studies, allows to investigate whole brain connectivity patterns without any task and with minimal assumptions compared to other approaches such as cardiac gating (52, 66). Given the findings of previous studies suggesting that CI is involved with autonomic nervous system structures (67–69), we hypothesized that the CI values would be correlated with brain regions belonging to the CAN, with higher CI values being predictive of greater positive correlations in this network.

## Methods

### Participants

This study included patients diagnosed as either UWS or MCS according to the Coma Recovery Scale - Revised (CRS-R) (14, 70) and diagnosed as either UWS or MCS who underwent an MRI examination under Propofol sedation together with electrocardiography (ECG) recordings. Exclusion criteria were (i) artifacts in ECG recording (ii) ECG acquisition and neuroimaging examination in patients less than 2 weeks from brain insult, (iii) large focal brain damage, i.e., >2/3 of one hemisphere, as stated by a certified neuroradiologist, (iv) motion parameters >3 mm in translation and 3 degrees in rotation. Additional exclusion criteria were applied for patients included in the MRI analysis: (v) suboptimal segmentation and normalization due to movement or metallic artifacts as stated by a certified neuroradiologist, (vi) non gaussian-like fMRI signal shape after denoising.

From an initial dataset of 67 sedated patients with ECG and imaging acquisition, 37 patients were discarded because of too many artifacts in the ECG recording. The 30 remaining patients formed the subgroup S1 with 14 patients (7 males, mean age 51 ± 14; 7 females, age 46 ± 18; 7 ARCA [cardiac arrest], 2 ANOX [anoxic], 1 TBI [traumatic brain injury], 2 HEM [hemorrhagic], 1 ANOX+TBI [anoxic and traumatic], 1 other [metabolic, epilepsy, etc.]) being diagnosed as UWS and 16 patients (10 males mean age 44 ± 17; 7 females, mean age 41 ± 17; all patients mean age 42 ± 17; 2 ARCA, 2 ANOX, 10 TBI, 1 HEM, 1 ANOX+TBI) as MCS (Table 1). For the correlation analysis between the CI values and brain regions connectivity differences as detected by resting-state fMRI, nine additional patients were discarded because of movement or metallic artifact in the fMRI data, or because of suboptimal segmentation or signal shape during the preprocessing as stated above (additional details are in the Supplementary Materials, Appendix B). The subgroup S2 for fMRI analysis therefore included 21 patients with 10 UWS patients (5 males, mean age 54 ± 11; 5 females, mean age 50 ± 18; 5 ARCA, 2 ANOX, 2 HEM, 1 ANOX+TBI) and 11 MCS patients (5 males, mean age 37 ± 17; 6 females, mean age 40 ± 16; all patients mean age 38 ± 16; 1 ARCA, 2 ANOX, 7 TBI, 1 HEM) (Table 1). The evolution time since the brain injury up to the ECG/MRI assessment is described in Table 1. The patients were matched between MCS and UWS for diagnosis, age, gender, etiology and onset, for both subgroups.

**Table 1:** Demographic information of patients.

**ID**	**CRS-R diagnosis**	**CRS-R total score**	**CRS-R subscore**	**Etiology**	**Age**	**Days since onset**
**1**	**UWS**	**3**	**S101100**	**OTHER**	**15–24**	**18**
**2**	**UWS**	**3**	**S001101**	**ANOX**	**55–64**	**21**
**3**	**UWS**	**3**	**S001101**	**ARCA**	**65–74**	**31**
4	UWS	4	S002101	ARCA	55–64	24
**5**	**UWS**	**4**	**S001201**	**ANOX**+**TBI**	**45–54**	**46**
6	MCS	5	S102101	TBI	15–24	38
**7**	**MCS**	**5**	**S030101**	**HEM**	**45–54**	**30**
8	UWS	5	S201101	ARCA	35–44	733
**9**	**UWS**	**5**	**S102101**	**ARCA**	**65–74**	**18**
**10**	**UWS**	**5**	**S002102**	**ARCA**	**65–74**	**43**
**11**	**MCS**	**6**	**S012102**	**TBI**	**15–24**	**31**
**12**	**UWS**	**6**	**S111102**	**ARCA**	**45–54**	**37**
13	UWS	6	S102102	HEM	55–64	248
**14**	**UWS**	**6**	**S101202**	**ARCA**	**45–54**	**101**
15	UWS	6	S111201	TBI	25–34	1017
**16**	**MCS**	**7**	**S302101**	**ARCA**	**45–54**	**209**
**17**	**MCS**	**7**	**S230101**	**TBI**	**25-34**	**534**
**18**	**UWS**	**7**	**S102202**	**HEM**	**45-54**	**353**
**19**	**UWS**	**8**	**S112202**	**ANOX**	**15-24**	**462**
**20**	**MCS**	**9**	**S311211**	**TBI**	**15–24**	**432**
**21**	**MCS**	**10**	**S232201**	**TBI**	**35–44**	**1294**
**22**	**MCS**	**10**	**S331102**	**ANOX**	**25–34**	**2407**
23	MCS	10	S115201	TBI	45–54	220
**24**	**MCS**	**11**	**S305201**	**TBI**	**25–34**	**561**
**25**	**MCS**	**11**	**S305102**	**ANOX**	**15–24**	**624**
26	MCS	12	S305202	TBI	15–24	660
**27**	**MCS**	**13**	**S335101**	**TBI**	**35–44**	**319**
28	MCS	15	S345102	ANOX+TBI	45–54	2086
29	MCS	16	S345202	ARCA	45–54	290
**30**	**MCS**	**16**	**S335212**	**TBI**	**55–64**	**4322**

*In bold: patients included in fMRI analysis (S2 group). Days since onset: evolution time since the brain injury up to the ECG/MRI acquisition. CRS–R subscore represent the subitems scores of the best CRS–R during the period of assessment (in order: “S” prefix for subscore then auditory, visual, motor, oromotor/verbal, communication and arousal scores). The rejection details for the patients discarded from the fMRI analysis are available in the Supplementary materials (Appendix B)*.

*ARCA, cardiac arrest; TBI, traumatic brain injury; HEM, hemorrhagic; ANOX, anoxic*.

The study was approved by the Ethics Committee of the Faculty of Medicine of the University of Liège and written informed consents, including for publication of data, were obtained from the patients' legal representatives and from the healthy control subjects in accordance with the Declaration of Helsinki.

### Sedation protocol

Patients were sedated to reduce the severity of movement artifact during the fMRI data acquisition. The sedation was obtained by Propofol infusion keeping the concentration to a minimum [average: 1.7 μg/mL, range: [1, 2.5] μg/mL] (71). The sedation was administered through intravenous infusion by a target-controlled infusion system [Diprifusor, pharmacokinetic model of Marsh et al. (72), Alaris TM, Alaris Medical Belgium B.V., Strombeek-Bever, Belgium] in order to obtain constant plasma concentration. Propofol was chosen for immobilization purpose for its short induction and recovery times, and because generally it does not need additional sedatives (73). Moreover is one of the most available anesthetic agent with common clinical application and well-established safety as well as being well-studied (74). There is also preliminary evidence that Propofol has also might not significantly reduce the residual resting-state functional connectivity observed in UWS and MCS patients (71). During data acquisition, the patients wore headphone and earplug. The stability of their vital parameters was controlled by continuous monitoring of blood pressure, ECG, respiration and pulse-oximetry.

### ECG procedure

#### ECG data acquisition

Electrocardiographic activity was recorded during the 10 min of fMRI data acquisition using the scanner's built-in equipment. The cardiac cycle was monitored by a photoplethysmographic sensor (PPG) placed on the right index finger and ECG's three leads positioned on the chest of the patients (leads I, II, and III are used and acquired in parallel via the ECG channels to display a prominent peak of the QRS ECG complex).

#### ECG data preprocessing

**Figure 1:**
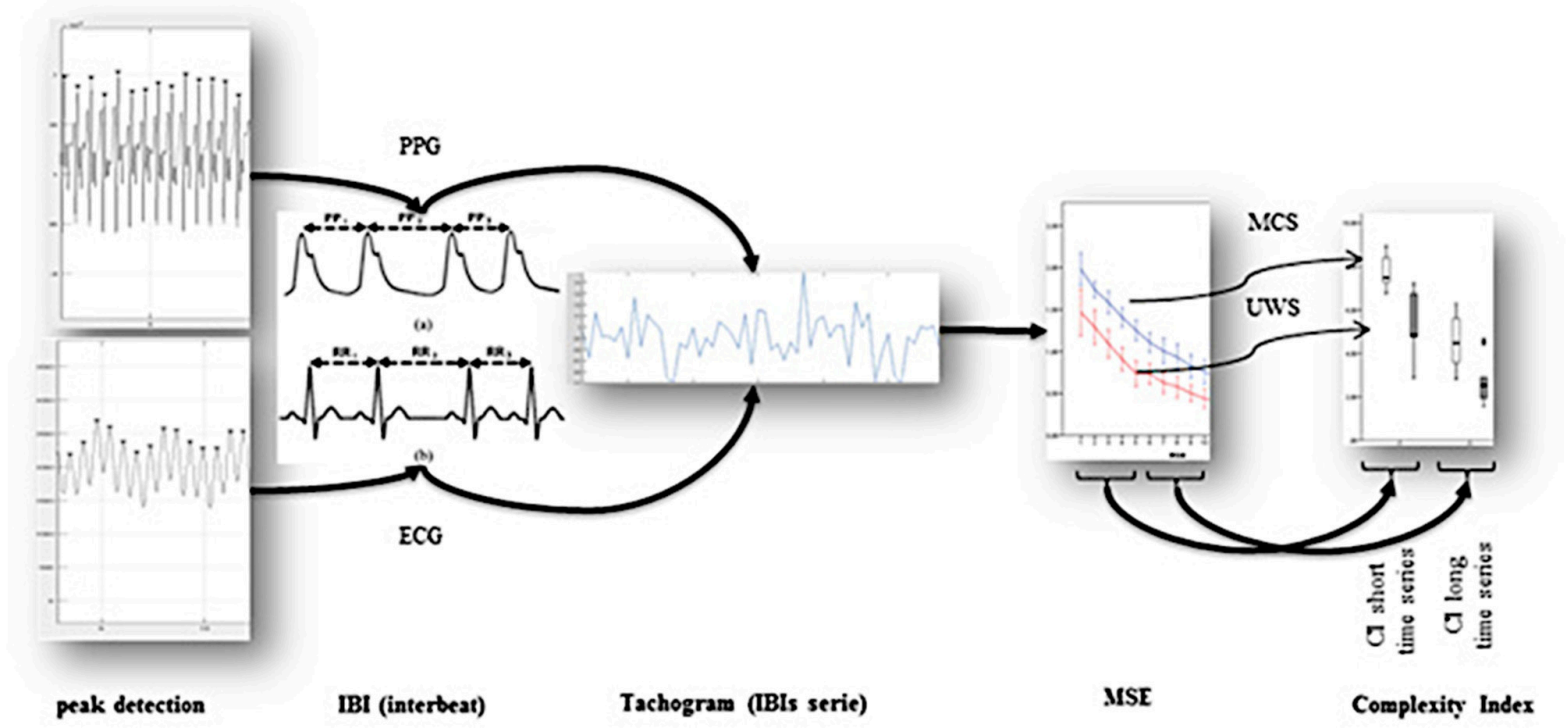
ECG/PPG data extraction and data analysis. First, the signal's QRS complex peaks are detected from ECG and PPG signals, from which the interbeat intervals are extracted. These intervals are used to produce an interbeat (IBI) series, showing in x axis the interval counter since the start, and as y-axis the duration of this interval. From this IBI series, the Sample Entropy is calculated over multiple time windows: first the standard Sample Entropy on each interval, then the Sample Entropy on the average of n intervals, allowing to compare the entropy of blocks of intervals instead of only the sudden change in between two consecutive intervals. Finally, these multiscale entropy values are averaged five by five into the Complexity Indices, one for the short time scale and one for the long time scale.

The ECG signal and PPG was cleaned of noise using a FFT filter without detrending (SigView software; http://www.sigview.com/). The series of consecutive intervals between heartbeats (tachogram) were extracted from ECG and PPG. After a visual analysis for ectopic beat or missing data, the MSE was calculated and analyzed to measure the complexity of the nonlinearity and non-stationary properties of the signal using the HRV Advanced Analysis software version 2.2 (75). Studies demonstrated that PPG and ECG measures have superimposable results in the temporal and frequency domains and in nonlinear dynamic analyses (76). The results between ECG and PPG signals were manually compared as an additional sanity check about the correct acquisition of the signal (Figure 1).

#### ECG data analysis

**Figure 2:**
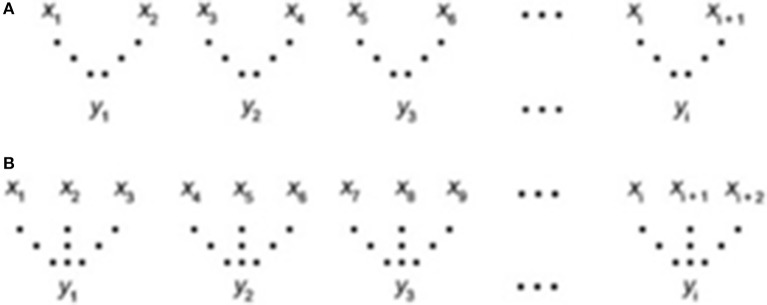
Coarse graining procedure. **(A)** scale 2, **(B)** scale 3, where the “x” series is the original IBI and the “y” is the new time series constructed through an averaging of the data points. For τ = 1 the course-grained scale is the original IBI sequence; A corresponds to the time series τ = 2, B corresponds to the time series τ = 3.

The MSE approach (38, 41) was applied to quantify the degree of irregularity over a range of time scales (τ). The method involves the construction of coarse-grained IBI time series and the quantification of the degree of irregularity of each of these. We then extracted 10 min from the tachogram. The time series from τ = 1–10 were constructed by averaging the IBI/tachogram's data points within non-overlapping windows of increasing length, τ (Figure 2).

Finally, the SE was applied for each coarse-grained constructed (37, 77) (Equation 1). The purpose of SE is to look for patterns in a time series and quantify its degree of predictability or regularity (77). The parameters involved in the calculation of the SE are the dimensional phase space *m* and the tolerance for accepting matches of two patterns *r* and were set to *m* = 2 and *r* = 0.15 (41, 78).

$$\begin{array}{l} {\text{S}}_{E}\left(m,r,N\right)=-\ln\;\frac{\phi^{m+1}\left(r\right)}{\phi^{m}\left(r\right)} \end{array}$$

Equation (1): SE: Sample Entropy; *m*: distance between time series points to be compared; *r*: radius of similarity; *N*: length of the time series; ϕ: probability that points m distance apart would be within the distance r.

The CI of the MSE is calculated as the area under the SE time scale curve (Equation 2).

$$\begin{array}{l} {\text{C}}_{I}=\overset{N}{\underset{i=1}{\sum }}S_{E}\left(i\right) \end{array}$$

Equation 2: CI summations of quantitative values of the Sample Entropy of N coarse-grained time scale.

The CI provides insights into the integrated complexity of a system, over a range of time scales of interest. The summations of quantitative SE values over time scales 1–5 and over time scales 6–10 represent the complexity index calculated in short (CI_s_) and long time scales (CI_l_), respectively (41), corresponding to high frequency (0.15–0.4 Hz) and low frequency band (0.04–0.15 Hz) respectively.

### MRI procedure

#### MRI data acquisition

All structural and functional images of the MCS and UWS patients were acquired on a 3 Tesla Siemens Magnetom TrioTim magnetic resonance image machine at the University Hospital of Liège.

#### Structural imaging

A high-resolution T1-weighted image was acquired for each patient (T1-weighted 3D gradient echo images using 120 slices, repetition time = 2,300 ms, echo time = 2.47 ms, voxel size = 1 × 1 × 1 mm^3^, flip angle = 9 degrees, field of view = 256 × 256 mm^2^) in order to allow for precise segmentation and coregistration as well as denoising.

#### Resting-state fMRI

Multislices T2^*^-weighted fMRI images were obtained during 10 min for each patient by using Echo Planar Imaging (EPI) sequence with axial slice orientation (300 volumes, 32 slices, voxel size = 3.0 × 3.0 × 3.75 mm^3^, repetition time = 2,000 ms, echo time = 30 ms, flip angle = 78°, field of view = 192 mm, matrix size = 64 × 64 × 32, delay = 0, slice order = sequential descending). As a standard protocol, all subjects were instructed to keep their eyes closed and not to think of anything in particular. Head motion was restricted by placement of a comfortable padding around each participant's head, and earplugs and headphones were placed on the patient's ears. The first three initial volumes were automatically discarded by the MRI scanner (dummy scans) to allow for longitudinal magnetization to reach steady-state (79).

#### MRI data pre-processing

##### Structural imaging

Structural (T1^*^-weighted) MRI images were manually reoriented to the anterior commissure/posterior commissure (AC-PC) scheme and then normalized and segmented into gray matter, white matter, cerebrospinal fluid, skull, and soft tissue outside the brain, using the “old segmentation” module and standard tissue probability map of Statistical Parametric Mapping 12 (SPM12) (www.fil.ion.ucl.ac.uk/spm).

##### Resting-state fMRI

Functional volumes were first manually reoriented and coregistered to the structural images, and then preprocessed by using SPM12 (SPM, RRID:SCR_007037). First, the EPI volumes were corrected for the temporal difference in acquisition among different slices using the slice timing correction module with the reference slice set to the first temporal slice, and then the images were realigned for head motion correction using a two-steps procedure: (1) realignment to the first volume and creation of the mean image, (2) then all images were realigned to the mean EPI image. The mean EPI image across all realigned volumes was then auto-coregistered to the structural image. Then the structural image was segmented into three tissues: gray matter (GM), white matter (WM), and cerebro-spinal fluid (CSF) in the subject's space, producing as a by-product of the segmentation the parameters of the transform from the subject's space to Montreal Neurological Institute (MNI) space. This transform was then used to normalize the structural image, the co-registered EPI images and the segmented tissues. Finally, all the coregistered and normalized EPI images were smoothed with an isotropic Gaussian kernel (8 mm full-width-at-half-maximum). A manual inspection of the whole BOLD timeseries motion was conducted from the SPM motion file to exclude any subject where the translational head displacement was greater than 1 mm, or if the rotational displacement was greater than 0.1 radians. With the aim of reducing loss of signal or whole subjects exclusion due to motion artifacts (80), we used the “scrubbing” technique from the ART toolbox (Artifact Detection Tools, RRID:SCR_005994)^1^ for artifactual volume detection and rejection using a composite motion measure (largest voxel movement) with a “liberal” threshold (global threshold 9.0, motion threshold 2.0, use scan-to-scan motion and global signal). With this approach, a volume was defined as an outlier (artifact) if the largest voxel movement detected was above the specified thresholds. We subsequently included outliers in the global mean signal intensity and motion as nuisance regressors (i.e., one regressor per outlier in the first-level general linear model). Thus, the temporal structure of the data was not disrupted. Several parameters were included in a linear regression using CONN v17F (Connectivity Toolbox, RRID:SCR_009550) and SPM12 to remove possible spurious variances from the data. These were (i) six head motion parameters obtained in the realigning step, (ii) scrubbing the outlier scans detected by ART's composite motion measure, (iii) non-neuronal sources of noise estimated using the anatomical component-based noise correction method [aCompCor; (81, 82)], which consists in regressing out the representative signals of no interest from subject-specific white matter and cerebro-spinal fluid, which were the top five principal components (PCA) from the white matter and the top five from cerebrospinal fluid per-subject mask (81). Then the residual time series were linearly detrended (no despiking) and temporally band-pass filtered (0.008–0.09 Hz) using CONN's denoising procedure.

### Statistical analyses

#### ECG statistical analyses

In both the entire patient group S1 and the subgroup undergoing fMRI analysis S2, the CI_s_ and CI_l_ measures average per MCS and UWS groups were compared using a Mann-Whitney exact test. Correlation between the CRS-R total score—the sum of all CRS-R items of the best assessment over a week—with the CI_s_ on one hand, and between the CRS-R total score and CI_l_ on the other hand was analyzed using the Spearman's correlation test. Significance of tests was set to p<0.05.

#### Machine learning model

WEKA (Waikato Environment for Knowledge Analysis, RRID:SCR_001214), an open source toolbox for machine learning analysis (64) ^2^ was used to assess the discriminative power of the CI measures by a machine-learning model called the One-R classifier (83), with the objective of predicting the CRS-R diagnosis of UWS or MCS given a patient's CI measures. The retained CRS-R diagnosis was the final best diagnosis over a week of CRS-R assessments. One-R (83) is a fast and very simple algorithm deriving a one level decision tree. It operates by generating a separate rule for each individual attribute of the dataset (CI_s_ and CI_l_) based on error rate. To generate the rule, each attribute is discretized into bins calculating the percentage that each class (MCS and UWS) appears within each bin. Finally, the rule for the final decision tree is chosen by selecting the attribute with minimum error to perform the diagnostic classification. This algorithm was chosen as it reported the best results in our case while being the most simple and thus robust model after running multiple simulations with various machine learning algorithms known to derive efficient models for diagnosis (84), the results of these simulations are available in the supplementary materials (Appendix A). The dataset used to generate the model consisted of the CI_s_ and CI_l_ values of the S1 group, and the objective was to predict the patient's diagnosis (UWS or MCS). To assess the performance of this model in generalization, a 10-fold cross-validation test (85) was conducted, thus the S1 group was split into 10 parts of equal number of patients, and the model was learnt on 9 parts and tested on the 10th part. This process was performed 10 times in total to use each part as the test set at some point, and metrics were calculated as the average over all 10 tests. Several metrics were calculated on both the 10-fold cross-validation test results, the S1 subgroup results and the S2 subgroup results such as the sensitivity (rate of MCS correctly classified), specificity (rate of UWS correctly classified), false positive and negative rates of MCS and UWS classification, accuracy (MCS and UWS predicted conditions), F1-score (86) [a measure of the test's accuracy that takes in consideration the harmonic mean of sensitivity and its precision also called the Dice similarity coefficient, ranging values between 0 [worst precision and sensitivity] and 1 [perfect precision and sensitivity]]and the Matthews Correlation Coefficient (87) [a correlation coefficient between the observed and predicted binary classifications, ranging values between 1 [perfect prediction], 0 [random prediction], and −1 [total disagreement between prediction and observation]].

#### Resting-state fMRI analyses

Functional magnetic resonance imaging is a non-invasive technique used to investigate the spontaneous temporal coherence in blood-oxygen-level dependent (BOLD) signal fluctuations related to the amount of synchronized neural activity (i.e., functional connectivity) existing between distinct brain locations (65)

With the aim of investigating the possible brain connectivity changes associated with a change of the CI values, we conducted a whole-brain resting-state fMRI functional connectivity analysis using a seed-to-voxel correlation analysis to observe changes in correlation of the BOLD signal in the whole brain with respect to the specified seed regions. Using CONN, we extracted from fMRI BOLD time series from a region of interest (the seed) and measured the temporal correlation between this signal and the time series of all other brain voxels. We have also conducted a voxel-to-voxel analysis by correlating the activity of all fMRI BOLD voxels to all other voxels via the Intrinsic Connectivity Contrast [ICC; in Conn toolbox; (88, 89)] as a quantification measure of global brain connectivity. In short, ICC quantifies the degree, including positive and negative correlations, of each voxel with all other brain voxels, which is then standardized against the average voxel degree as the mean and variance 1 to derive a Z-score. In other words, a positive ICC means that a brain region is significantly more connected to the rest of the brain compared to the average voxel connectivity.

The seeds were defined as spheres of 5 mm radius around the peak coordinates of main structures of the ANS/CAN (90): the Superior Temporal Gyrus (STG) [−44, −6, 11] & [44, −6, 11], the Dorso-Lateral PreFrontal Cortex (DLPFC) [−43, 22, 34] & (22, 34, 42), the Fronto-Insular cortex (FI) [−40, 18, −12] & [42, 10, −12], the Paracingulate cortex (PC) [0, 44, 28], the anterior cingulate cortex/mesioprefrontal cortex (ACC/MPFC) [−1, 54, 27], the posterior cingulate cortex/precuneus (PCC/precuneus) [0, −52, 27], cerebellum [−4, −56, −40], thalamus [−4, −12, 0],[4, −12, 0]. Their coordinates have been taken from previous studies in order to avoid circularity (16). We used the averaged time series to estimate whole brain positive correlation r maps, and the *t*-test contrasts. In the design matrix, we applied a contrast to regress out the average connectivity of MCS and UWS patients and to highlight any connectivity difference that is correlated only with the complexity index. We did two different correlation tests for CI_s_ and CI_l._

Finally, we examined global brain connectivity patterns (without a priori seed) between each voxel and the rest of the brain using the ICC measure. We used the same design matrix to highlight only the connectivity differences correlated only with CI_s_ and then CI_l_.

Age standardized to unitary standard deviation and centered to the mean was used a regressor of nuisance in the design matrices for both the seed-based and the hypothesis-free analyses.

Statistical results were generated with CONN and considered significant with multiple comparison correction at the topological level with non-parametric permutation test cluster-mass p-FWE < 0.1 and with primary voxel-wise threshold p-uncorrected < 0.001 with 1000 iterations. CONN 17f was patched with a permutation test patch to allow for generalized permutation of residuals (https://www.nitrc.org/forum/message.php?msg_id=23131). The significant regions names were derived from the Harvard-Oxford atlas (Harvard - Oxford Cortical Structural Atlas, RRID:SCR_001476), using bspmview tool ^3^ Visualizations were generated using CONN, MRIcron (RRID:SCR_002403), NiLearn (RRID:SCR_001362) (91), Python (Python Programming Language, RRID:SCR_008394) and an in-house python script (https://github.com/lrq3000/neuro-python-plotting).

## Results

**Figure 3:**
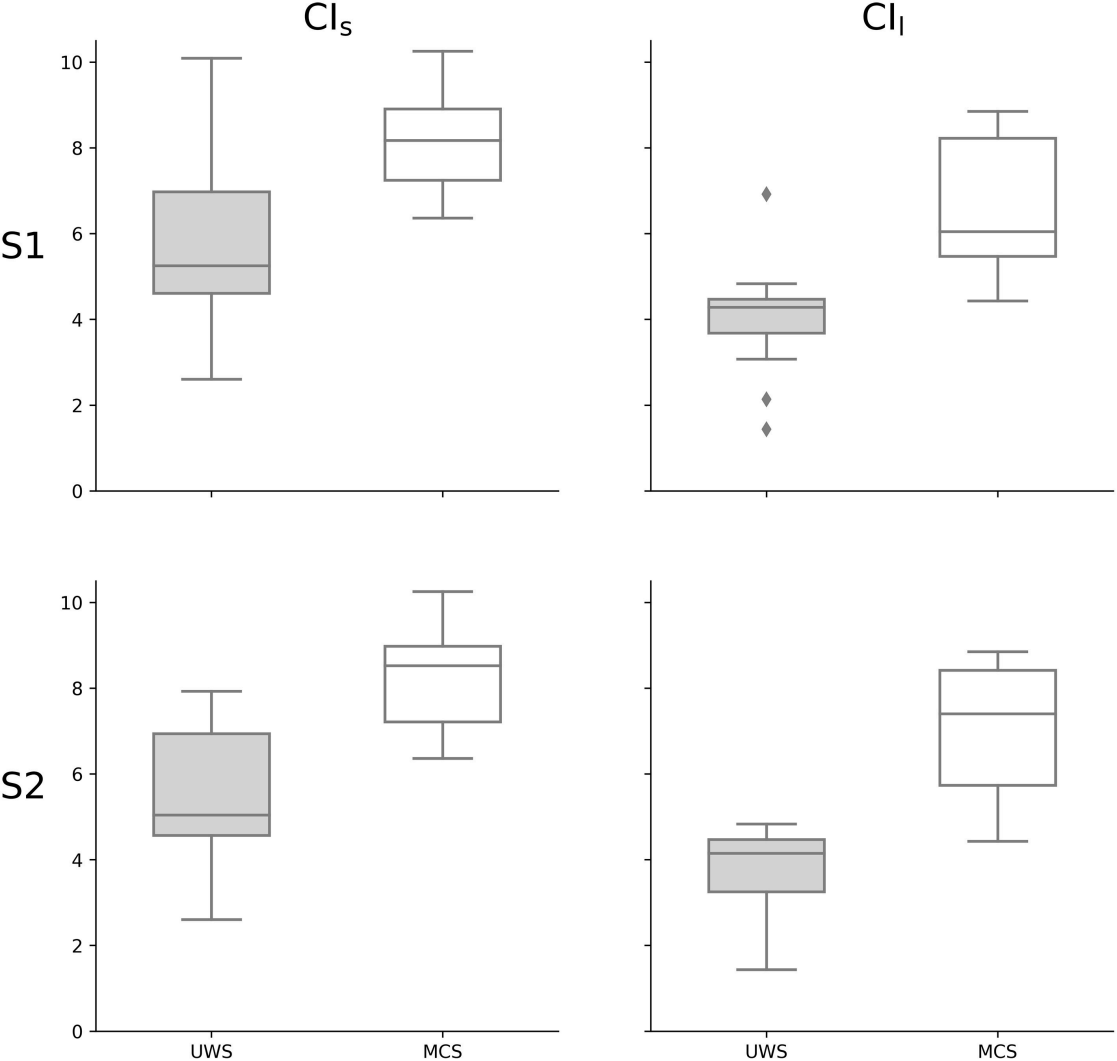
Complexity Index statistical analysis comparing UWS and MCS patients summarized as a box plot. Higher values of CI_s_ (*z* = −3.346, *p* < 0.001) and of CI_l_ (*z* = −4.095, *p* < 0.0001) were observed for MCS group compared to UWS using Mann–Whitney's test. The 1st row compares the entire group of patients S1 (*n* = 30), while the 2nd row compares the subgroup of patients S2 (*n* = 21) who underwent fMRI analysis. The 1st column represents the complexity index (CI) in short time scale, while the 2nd column is for the long time scale. White boxes represent MCS patients; gray boxes the UWS patients. The boxes range from Q1 to Q3, while the whiskers are defined at the 1.5 interquartile range, and the black lines are the medians, points are outliers.

In the S1 group, when comparing the CI values of MCS and UWS patients, higher values of CI_s_ (*z* = −3.346, *p* < 0.001) and of CI_l_ (*z* = −4.095, *p* < 0.0001) were observed for the MCS group compared to the UWS group (Figures 2, 3). A stronger correlation was found between the CRS-R total score and CI_l_ (Spearman's rho = 0.671, *p* < 0.0001) compared to the moderate correlation between CRS-R total score and CI_s_ (Spearman's rho = 0.579, *p* < 0.001) (**Figure 5**). The results of the S1 group are superimposable to the subgroup S2 who underwent fMRI analysis. In the S2 subgroup, higher values of CI_s_ (*z* = −3.063, *p* = 0.002) and of CI_l_ (*z* = −3.556, *p* < 0.001) were observed in the MCS group compared to the UWS group (Figure 3). A stronger correlation was found between the CRS-R total score and CI_l_ (Spearman's rho = 0.676, *p* < 0.001) compared to the moderate correlation between CRS-R total score and CI_s_ (Spearman's rho = 0.619, *p* = 0.003) (**Figure 5**).

Using the machine learning One-R classifier, the CI_l_ was selected as the most discriminating feature for the diagnostic classification of MCS and UWS patients. The model's accuracy in the classification of MCS and UWS patients was 93%, with a correct classification of MCS and UWS of 94 and 93% respectively (Table 2). The false positive (UWS as MCS) and false negative (MCS as UWS) rates were 7 and 6% respectively. F1-score and Matthews Correlation Coefficient were 94 and 0.87 respectively, evidencing a high performance of the model in the diagnostic classification. Superimposable results were obtained in the 10-fold cross-validation test (Table 2), with an accuracy of 90% and a correct MCS and UWS classification of 88 and 93% respectively. The false positive and false negative rates were 7 and 13% respectively.

**Table 2:** One–R classifier results and confusion matrix.

**Confusion Matrix**	**Classifier: One-R**		
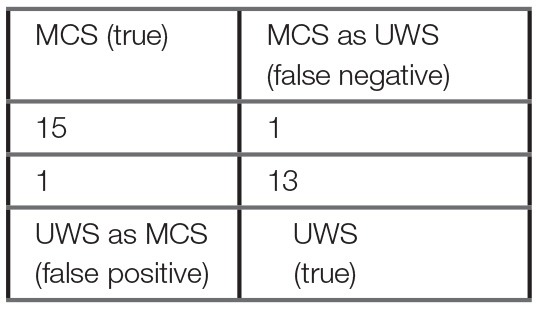	Rule: CI_l_ < 4.876 → UWS CI_l_ ≥ 4.876 → MCS		
	Test dataset results		
	Full training test (S1 group)	10-fold cross-validation	fMRI test (S2 subgroup)
True positive (MCS) rate (%)	94	88	92
True negative (UWS) rate (%)	93	93	100
False negative rate (%)	6	13	8
False positive rate (%)	7	7	0
Precision MCS classification (%)	94	94	100
Precision UWS classification (%)	93	87	91
accuracy (%)	93	90	95
F1-score (%)	94	90	96
Matthews Correlation Coefficient[−1:1]	0.87	0.80	0.91

*The confusion matrix is based on the S1 group. The One-R classifier is a simple machine learning decision tree model that derives a single rule from the single most contributing parameter to predict the patient's diagnosis. This model deduced that the long term complexity index (CI_l_) is the best predictor of patient's diagnosis, with a threshold of ~4.9, below which the patient should be diagnosed as unresponsive (UWS) and above as minimally conscious (MCS). The 10-fold cross-validation test shows that this model is quite robust and reliable, with 90% accuracy, 7% false positive rate, 13% false negative rate and a F1-score, combining both accuracy and recall, of 90%. For comparison, a baseline Zero-R rule always predicting MCS as the diagnosis would have an accuracy of 53% on the S1 group dataset. Additional machine learning models and results can be found in the Supplementary materials (Appendix A)*.

These results showed that most MCS patients displayed more complex HRV patterns compared to UWS patients. In addition, the CI measures showed strong discriminative power when used to predict the diagnosis of a patient. Under the frame of the brain-heart two-way interaction and with the aim to observe how this complexity is linked to the brain activity, we investigated the resting state fMRI of a subset of 24 patients who had sufficient image quality to ensure successful analysis. We chose to focus on only positive correlations, using one-sided statistical test and multiple comparison correction at the cluster level with non-parametric permutation test (**Figure 6**). Both CI were positively correlated with an increase of the brain's functional connectivity in CAN regions. Increased values of CI_s_ were associated with increased connectivity between the Fronto-Insular cortex with the Superior Frontal Gyrus and between the Paracingulate cortex with two clusters covering the inferior and middle Temporal Gyrus, the Frontal Operculum and the Insular cortex. CI_l_ values positively correlated with an increase of connectivity between the Paracingulate cortex with the right Frontal Pole, between the Superior Temporal Gyrus (STG) with the Superior Parietal Lobule (SPL) and finally between the Dorso-Lateral PreFrontal Cortex (DLPFC) located in the Middle Frontal Gyrus (MFG) with the left and right Frontal Pole. The Anterior Cingulate Cortex, the Medial Prefrontal Cortex, the Thalamus and the Cerebellum did not show significant results. Statistical tables are available in the Supplementary materials (Appendix B).

The ICC showed a positive correlation between the CI_s_ and the intrinsic connectivity (i.e., an overall connectivity with the rest of the brain) in a cluster covering the Middle Temporal Gyrus (MTG) and the STG and between the CI_l_ and the intrinsic connectivity of the MFG. Of interesting note, both the seed-based and the hypothesis-free analyses found an increase of connectivity in the STG and MFG correlated with an increase of CI. By comparing only the functional connectivity of MCS to UWS patients, without CI measures, no significant results were found except for the ICC analysis (see the Supplementary materials, Appendix B).

## Discussion

We investigated the HRV and more specifically the CI of the MSE in MCS and UWS sedated patients, tested its discriminative power for diagnosis and investigated the possible neural correlates sources of CI modulation via a resting-state fMRI analysis. The present study is the first to show that baseline HRV entropy, more specifically the CI, can be a reliable predictor of the clinical level of consciousness, and furthermore the first to estimate the direct relationship between CI and CRS-R and between CI and the brain functional connectivity using simultaneously acquired resting-state fMRI.

**Figure 4:**
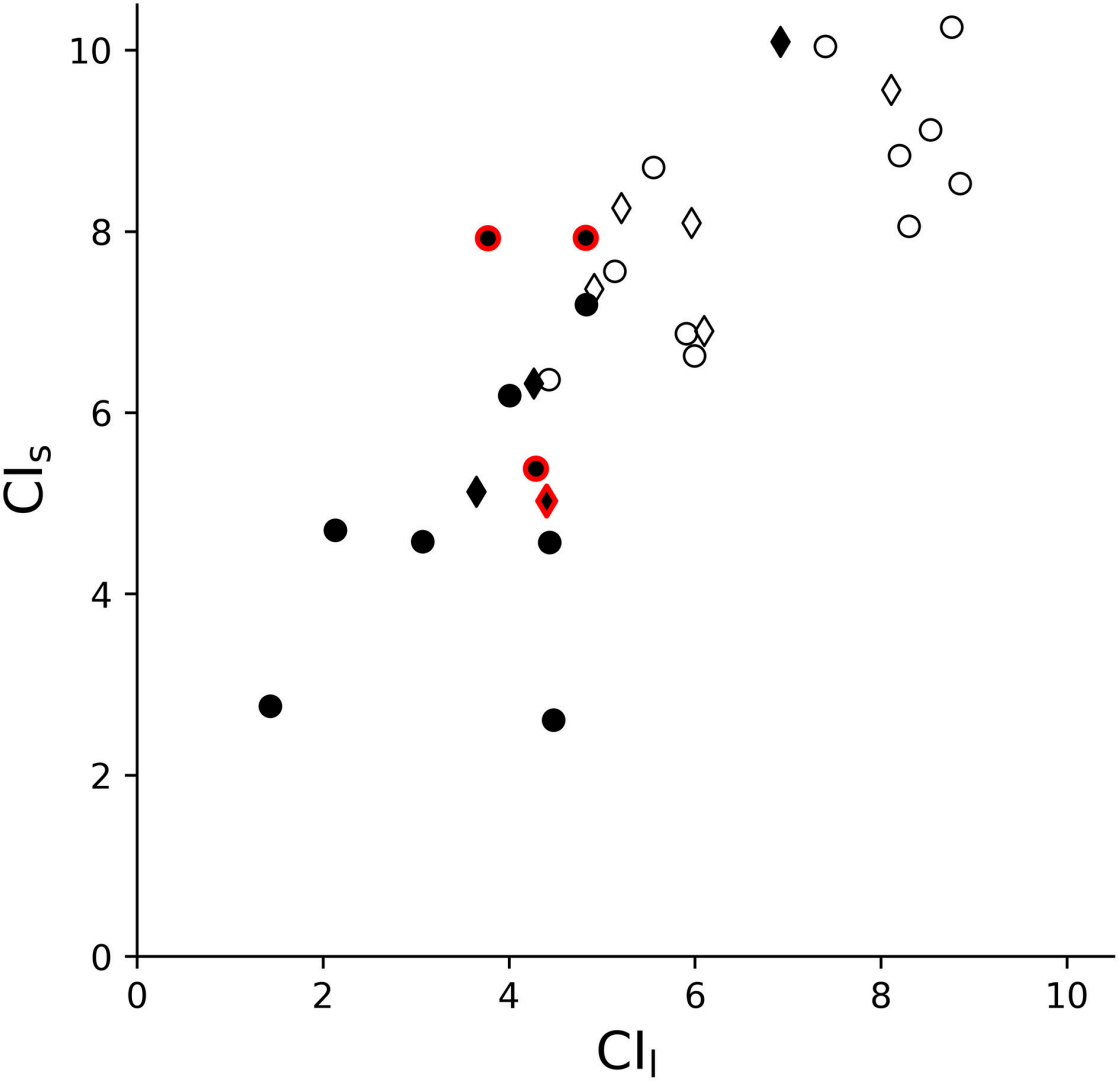
Dispersion graph of CI_l_ and CI_s_. This shows that the repartition of patients relatively to the CI is defined by the diagnosis, with UWS patients usually on the lower-end and MCS patients on the higher-end, showing some degree of linear separability. White circles and diamonds represent MCS patients; black circles and diamonds the UWS patients. Diamonds represent the patients discharged for the fMRI analysis (i.e., only included in S1, *n* = 30) while the circles are the patients included in the fMRI analysis (S2 group, *n* = 21). Outlined in red are patients in subacute state (i.e., with MRI acquisition between 2 and 4 weeks from brain insult).

**Figure 5:**
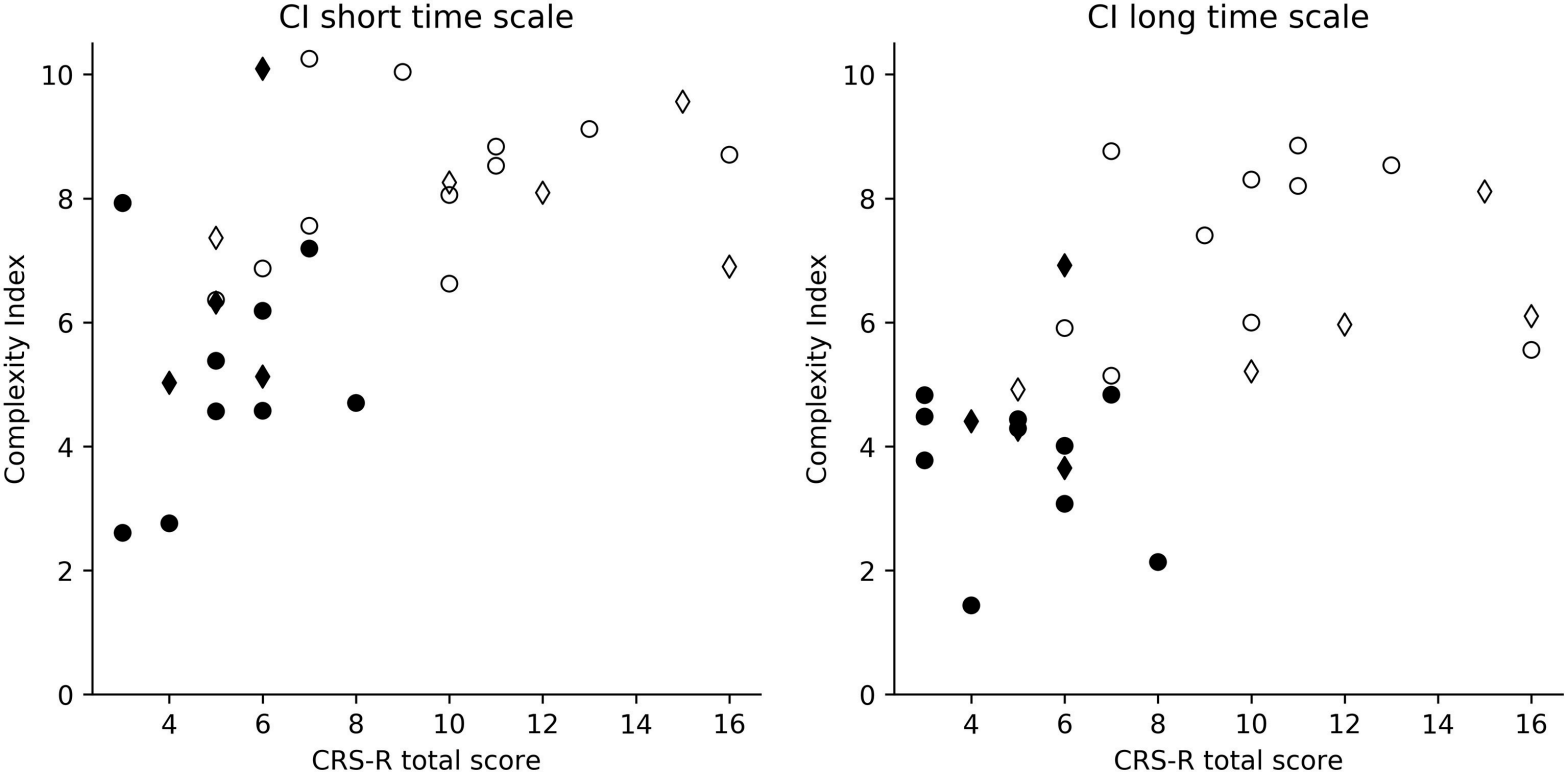
Dispersion graphs of the correlation between CRS-R total score and CI. This shows the per-subject CI value relatively to the patient's CRS-R total score. Both CI_l_ (Spearman's rho = 0.671, *p* < 0.0001) and CI_s_ (Spearman's rho = 0.579, *p* < 0.001) were correlated with the CRS-R total score. S1 (diamond and circle markers, *n* = 30) and S2 (circle markers, *n* = 21) groups were compared for CI_s_ (left) and CI_l_ (right). White circles represent MCS patients; black circles the UWS patients. Diamonds represent the patients discharged for the fMRI analysis while the circle markers represent the patients included in the fMRI analysis (S2 group).

Group-wise, we found higher values of CI in MCS patients compared to UWS patients (Figure 3). This difference was observed for both the CI_s_ (linked to the parasympathetic modulation) with moderate significance and the CI_l_ (linked to the sympathetic modulation) with strong significance. Moreover, the values of CI were correlated to the CRS-R total score (Figure 5), with MCS patients generally displaying a higher-end CI value compared to UWS patients, with only UWS patients having CI values in the lower-end (Figures 4, 5).

To assess the discriminative power of CI for disorders of consciousness, we built a machine learning model based on the One-R rule association algorithm, using both CI as input features, with the objective to predict whether a patient is MCS (positive condition) or UWS (negative condition). The One-R algorithm derives a single rule from the single most contributing parameter to predict the patient's diagnosis. This classifier deduced that CI_l_ was the best predictor of patient's diagnosis, with a threshold of ~4.9, below which the patient should be diagnosed as UWS and above as MCS. According to the best standards in machine learning for neuroimagery, we conducted a 10-fold cross-validation test to evaluate the generalizable performance of this model (85) (Table 2), which showed that this model is quite robust and reliable, with 90% accuracy, 7% false positive rate, 13% false negative rate and a F1-score, combining both accuracy and recall, of 90%. For comparison, a baseline Zero-R rule always predicting MCS as the diagnosis would have an accuracy of 53% on the S1 group dataset. Thus, the model reported a high accuracy performance, while having low false positive and negative rates compared to the CRS-R gold standard. Since the One-R model is a very simple classifier with a linear decision frontier based on only one feature, this suggests that CI_l_ is a highly discriminative measure for UWS and MCS. Considering the much higher misdiagnosis rate of about 40% by human assessors not using the CRS-R, even after nation-wide efforts to reduce it (12, 13), and considering the very simple machine learning model used here, these results strongly suggest that heart rate CI might have an application as a complementary assessment tool and might help physician in their decision process by providing a supplementary hypothesis-free evaluation of the patient's state of consciousness.

**Figure 6:**
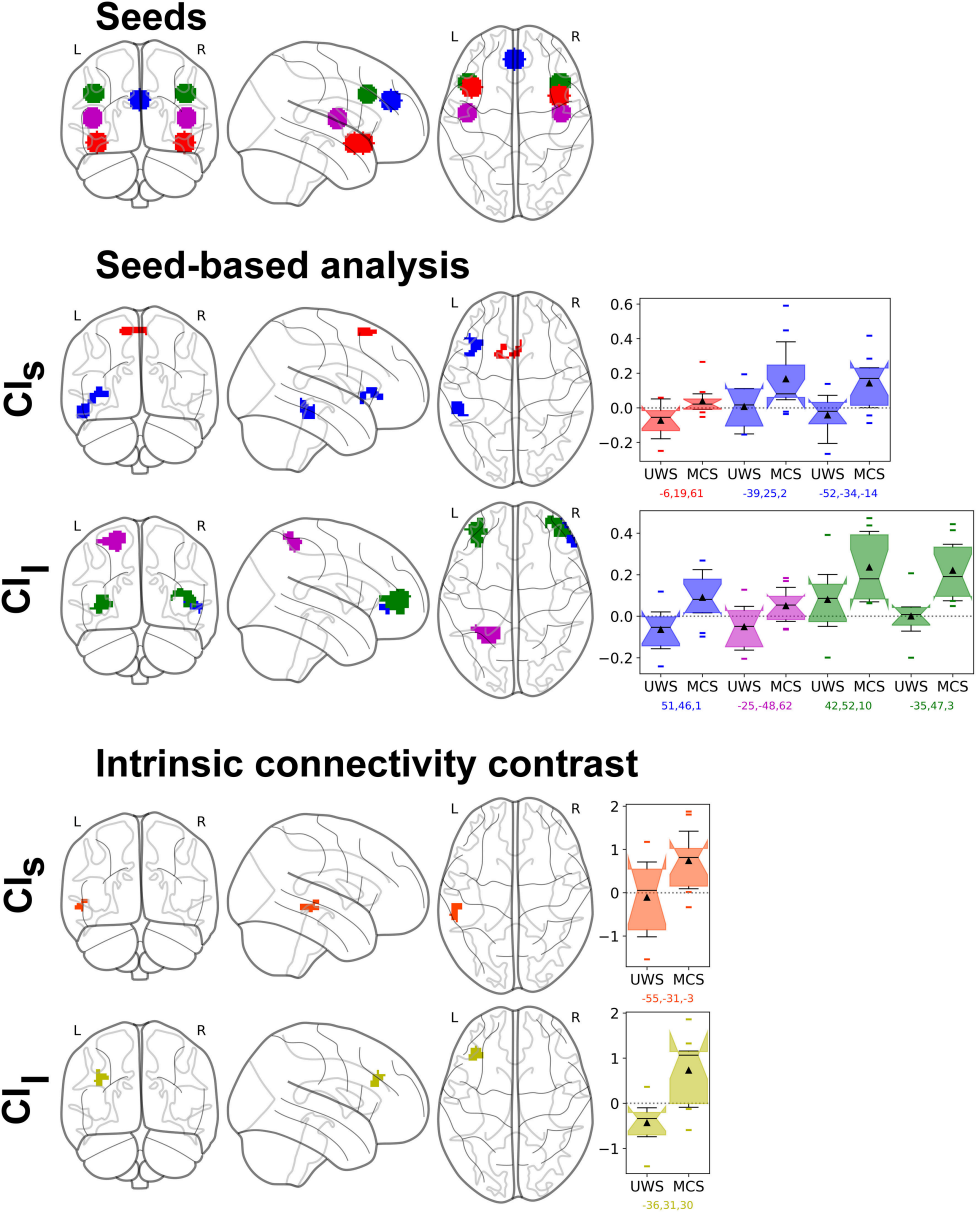
Resting-state fMRI analysis results of the parametric regression between CI and UWS/MCS patients' connectivity changes in the S2 group (*n* = 21). Top row shows the seeds: Fronto-Insular (FI, red), Paracingulate cortex (PC, blue), Superior Temporal Gyrus (STG, magenta), Dorso-lateral prefrontal cortex (DLPFC, green). Middle rows show the seed-based analysis results, with same colors as the seeds, and effect size as box plots (range Q1-Q3, whiskers interquartile 1.5, black line as median, black triangle as mean, points as outliers), first with the CI in short time scale (CI_s_) and then long time scale (CI_l_). We can see a positive correlation of the CI_s_ with the connectivity between FI with Superior Frontal Gyrus (red) and between PC with two clusters covering the Temporal Gyrus (inferior and middle), the Frontal Operculum and the Insular Cortex (blue). The CI_l_ is positively correlated with the connectivity between PC and the right Frontal Pole (blue), between STG with the Superior Parietal Lobule (magenta) and between DLPFC and the left and right Frontal Poles (green). Bottom rows show the hypothesis-free intrinsic connectivity correlation (ICC) results, with a positive correlation between values of CI_s_ and an increase of intrinsic connectivity of the posterior Middle Temporal Gyrus (pMTG) and posterior STG (orange); and a correlation between CI_l_ and an increase of intrinsic connectivity in the Middle Frontal Gyrus (MFG) (yellow). Statistical significance was considered at permutation of residuals test cluster-mass p-FWE < 0.1 and primary threshold *p*-uncorrected < 0.001.

Finally, the fMRI analysis reported a positive correlation between the CI and the connectivity in several brain areas belonging to the CAN/ANS (Figure 6), using both seed-based, thus guided, approach and voxel-based, thus hypothesis-free, approach. Indeed, the voxel-based ICC results showed that, even without any a priori about the spatial location of connectivity changes associated with higher CI values, we could observe that higher CI values were associated with brain regions belonging to the CAN/ANS.

Many studies have reported the potential usefulness of HRV analysis (in both time and frequency domains, as well as non-linear analysis) in consciousness studies (18, 19, 92). They observed better autonomic response to specific stimuli (i.e., music, visual, acoustic), higher sympathetic activation, modulation in peak of the low frequency band or ratio between low and high frequency power in MCS than in UWS (93–101). A greater HRV responsiveness in time and frequency domains to emotional stimuli than to non-emotional stimuli has been observed in MCS patients compared to UWS (97) and similarly for nociceptive stimuli (92) and auditory oddball tasks (102).

In the frequency domain, modulation of sympathetic response (observed by the normalized unit of low frequency) has been associated to musical stimuli (selected to elicit specific emotional response) in UWS patients (95), MCS patients and healthy subjects (93), allowing the experimenters to classify the subjects' emotional responses as positive or negative. For acute traumatic patients, pre-hospital low entropy has been associated with mortality, independently of GCS score or Injury Severity Score (103). MSE measured within the first 24 h can identify trauma patients at increased risk of subsequent hospital death (69) and predict robustly within 3 h of admission the death of the patients occurring days later (104). SE has proved useful for rapid identification of trauma patients with potentially lethal injuries (105). In pediatric patients, the reduction of heart rate dynamics was shown to correlate negatively with disease severity and outcome (106).

However, few studies have reported results in the non-linear domain (i.e., approximate entropy, sample entropy, multiscale entropy, etc.) in chronic patients with disorders of consciousness. In these few studies, lower values of approximate and sample entropy have been reported in UWS than MCS patients compared to healthy subjects following musical stimuli with increasing structural complexity (107, 108). Studies with anesthetized healthy subjects have reported decreased entropy during anesthesia (109, 110). Decreased sample entropy and approximate entropy values have also been reported in UWS and MCS compared to healthy subjects (103, 105).

We here investigated the HRV of mostly chronic patients with disorders of consciousness by using the MSE, which is a non-linear analysis that can capture a wider dynamic range of interaction between heart and brain than simple entropy or variability in the linear (time) or spectral (frequency) domains, and therefore potentially bear more diagnostic and prognostic information.

Indeed, cardiovascular signals are largely analyzed using traditional time and frequency domain measures, however these measures are not capable of measuring dynamic changes in the autonomic control of the heart rate, thus failing to account for important properties related to multiscale organization and brain-heart non-equilibrium dynamics (111–113).

The brain-heart dynamic processes, that characterize the cardiac signal output, can be described as non-linear, non-stationary, asymmetric and with multiscale variability (i.e. small perturbation can cause large effects, the system's output has dynamical properties that can change over time, the system dissipates energy as it operates far-from-equilibrium, and exhibits spatio-temporal patterns over a range of scales) (114).

In contrast, these dynamic processes in healthy conditions exhibit complex fluctuations that are reduced or absent in pathological conditions, where we can observe less complex outputs (115) expressed by an increased randomness (e.g., in a subject with atrial fibrillation) or augmented periodicity (e.g., in UWS patients).

Our results in the non-linear domain showing higher CI in MCS than UWS are in line with the above-cited literature and further characterize the complexity of brain-heart interactions. Our findings are also highly significant compared to previous studies using other types of analysis (100, 107, 108, 116). This confirms that the extra information extracted using non-linear analyses can lead to better differential diagnosis with high discriminative power, even higher than that of the clinical consensus without CRS-R (13), which can potentially be applied to clinical practice in a near future.

Several fMRI studies on healthy subjects have shown the complexity of interaction of the heart with the Central Autonomic Network (22, 52, 53, 117, 118). Valenza and colleagues have shown that the insular cortex, frontal gyrus, lateral occipital cortex, paracingulate and cingulate gyrus and precuneus cortices, as well as subcortical structures including the thalamus are involved in the modulation of the CAN/ANS network-mediated cardiovascular control (119). The causal, directed interactions between brain regions at rest (brain-brain networks) and between resting-state brain activity and the ANS outflow (brain-heart links) have been studied by Duggento et al. (120) showing that the amygdala, hypothalamus, brainstem and, among others, medial, middle and superior frontal gyri, superior temporal pole, paracentral lobule and cerebellar regions are involved in modulating the CAN. Previous studies reported that CI_s_ is probably linked to the vagal control of HRV, while CI_l_ seems to be more related (although not exclusively) to the sympathetic control of HRV (41, 78, 121, 122).

While most of these studies used active tasks paradigms or cardiac gating to investigate HRV (52, 66), the fMRI results of our study extend the previous findings by offering a new approach with two innovations: (1) by studying the resting-state connectivity changes, after the regression of physiological noise by principal components analysis via aCompCor, rather than by using an active paradigm or cardiac gating, which allows to estimate how the CI relates to the baseline cognitive abilities of the patient; (2) by investigating the direct correlation between the heart rate's complexity modulation (as measured by the CI) with the brain areas connectivity in regions involving the autonomic system, in order to identify some of the cerebral sources of HRV modulation. We found that both CI_s_ and CI_l_ are linked to the brain's functional connectivity of the CAN/ANS, with higher CI values being correlated with a recovery of CAN/ANS faculties. Indeed, by looking at the effect sizes, we can observe that the correlation is positive in MCS and usually close to null for UWS, suggesting a recovery of real positive connectivity in MCS as compared to UWS. Of note, we observed that the DLPFC, which seem to be a component of the CAN unique to humans (52, 53, 123), had a greater connectivity with the Frontal Poles correlatively with the CI_l_.

This highlights that (impaired) complex brain-heart interactions characterize chronic patients with disorders or consciousness, and that the CI can reflect these connectivity changes at resting state, in the form of a scalar value summarizing the connectivity changes of multiple regions of the CAN/ANS. This further suggests that the CI could potentially be used as a fast, inexpensive and entirely non-invasive method of screening and monitoring connectivity changes in the CAN/ANS networks. Combined with the observation of a high discriminative power using a model as simple as the one rule association of the One-R machine learning model, CI could represent a very interesting alternative for medical centers that cannot afford expensive MRI machines as well as for highly busy medical centers as a preliminary screening method. Furthermore, this method can work even for patients with extensive brain damages that might prevent neuroimagery methods from functioning.

Although less practical and affordable than ECG, future studies should investigate whether screening directly the functional connectivity change patterns in the CAN might also yield predictive value for the diagnosis, although we expect with less sensitivity than the CI. Indeed, our fMRI results suggest that the CI measures reflect an aggregation of various functional connectivity changes in the CAN, which allows for increased sensitivity compared to any single seed analysis.

Interestingly, one of the three UWS patients with a high CI evolved into a MCS state one year later after the assessment considered here. The CI measures might prove clinically relevant not only for diagnosis but also as outcome predictors. Future studies to assess the prediction power the CI measures are warranted.

This study is however not free of limitations. As patients suffering from disorders of consciousness notably move a lot (e.g., spasms, spasticity) and since fMRI data are very sensitive to movement, Propofol was here used in low doses in order to avoid movement artifacts during the fMRI scan acquisition, as required by clinical practice. HRV entropy is known to be profoundly affected by general anesthesia and it can play more roles in the monitoring of anesthetic depth (124). SE decreases after induction of anesthesia (110) and decrease of HRV entropy following Sevoflurane and Propofol anesthesia (109) has been observed. However, there is preliminary evidence that sedation might not exert a significant influence on the resting-state functional connectivity of UWS and MCS patients, since the impairment following the brain injury somehow overshadows the sedation effect (71, 125).

The ECG used in this study was acquired simultaneously to MRI, as was the standard procedure at the time at the Hospital of Liège. It would however be interesting for future studies to additionally acquire ECG outside of MRI acquisitions, which would be useful to derive additional metrics and assess the possible influence of MRI auditory noise on resting-state ECG. Indeed, a previous work observed that MCS patients show a phase shift of their cardiac cycle to global regularities in auditory signal (102), thus it is conceivable that the auditory noise induced by a MRI machine might impact the ECG.

Recently, there were a few findings about the circadian rhythm and body temperature fluctuations in disorders of consciousness, finding that several parameters such as the HRV, the body temperature and the circadian rhythm are correlated with the prognosis (126, 127). Furthermore, the preliminary results from an ongoing work investigating the day-to-night variations of the HRV in disorders of consciousness seem to indicate that the circadian cycle impacts directly the HRV, with more difference between groups being highlighted during the day. If this is confirmed on a bigger sample, this would indicate that ECG acquisition should be preferentially done during the day, as was done in our study (128).

Although the difficulty to recruit and analyze such a challenging population of patients should be noted, the relatively limited number of patients, heterogeneity of their etiology and time of disease onset can represent a limit for this study. For instance, outcome studies have highlighted that there is a correlation between the etiology and the final diagnosis (129). Due to the heterogeneity of our cohort of patients, a characterization of etiology is not possible. Future studies with a larger cohort of patients are needed to evaluate the relationship between the heart rate CI measures and the etiologies. The CI_l_ threshold found by the OneR classifier seems to be quite stable according to the 10-fold cross-validation test, but this threshold should be confirmed in practice on a larger population and on multiple centers in order to account for inter-scanners variability. Furthermore, we used the CRS-R diagnosis as the gold standard for most analyses and notably machine learning, which, like other behavior-based clinical assessment methods, might produce false negative errors as explained in the introduction, as previous studies observed UWS patients retaining covert consciousness (13, 130, 131). Finally, for the fMRI analysis, the CI, being based on the HRV, correlation with brain regions connectivity results might be partly influenced by blood irrigation variation.

## Conclusion

Our findings show that the MSE analysis of HRV and in particular the CI could be a useful tool to measure the degree of complexity in the brain-heart interaction and the response of the CAN/ANS systems to external stimulations. With the CI being correlated and even predictive of the clinical level of consciousness as assessed by the CRS-R, this could represent a fast, effective, inexpensive, and particularly easy to use tool to evaluate the level of consciousness in patients with disorders of consciousness. In particular, our findings show that CI has potential to be a useful supporting metric in the differential diagnosis between UWS and MCS, as well as a way to monitor patients' consciousness and brain connectivity evolution, in particular with patients that cannot be assessed with neuroimagery because of artifacts or extensive brain damage.

## Author contributions

FR and SKL contributed equally to this work. FR and SKL conceived, planned, and conducted the research and analyses. FR, SKL, and CD interpreted the results. FR designed the methods and scripts for data preprocessing and analysis on ECG and SKL and MB designed the methods and scripts for data preprocessing and analysis on fMRI. FR and SKL realized the visualizations. LH, CM, MC, VC-V, CA, AV, and CC carried the data acquisitions. CD provided guidance and supervision on the whole study. CD, SL, and CC provided supervision and resources. FR, SKL, and CD drafted the manuscript and all authors provided critical feedback and helped shape the final manuscript.

### Conflict of interest statement

The authors declare that the research was conducted in the absence of any commercial or financial relationships that could be construed as a potential conflict of interest.

## Footnotes

^1^*NITRC: Artifact Detection Tools (ART): Tool/Resource Info*. Available at: https://www.nitrc.org/projects/artifact_detect/ (Accessed March 1, 2018).

^2^*Weka 3 - Data Mining With Open Source Machine Learning Software in Java*. Available online at: https://www.cs.waikato.ac.nz/ml/weka/ (Accessed April 27, 2018).

^3^BSPMVIEW|bspmview. Available online at: http://www.bobspunt.com/bspmview/DOI 10.5281/zenodo.168074 (Accessed March 1, 2018).

## Abbreviations

ACC: anterior cingulate cortex
AC-PC: anterior commissure - posterior commissure
ANOX: anoxic
ANS: autonomic nervous system
ARCA: cardiac arrest
BOLD: blood-oxygen-level dependent
CAN: central autonomic network
CI: complexity index
CI_l_: complexity index in the long term (average of multiscale entropies from 6 to 10)
CI_s_: complexity index in the short term (average of multiscale entropies from 1 to 5)
CNS: central nervous system
CRS-R: Coma Recovery Scale – Revised
CSF: cerebro-spinal fluid
ECG: electrocardiogram
EPI: echo-planar imaging
FFT: Fast Fourier Transform
fMRI: functional magnetic resonance imagery
GM: grey matter
HEM: hemorrhagic
HRV: heart rate variability
ICC: intrinsic connectivity contrast
LOC: Lateral Occipital Cortex
MCS: minimally conscious state
MFG: middle Frontal Gyrus
MNI: Montreal Neurological Institute
MPFC: Medial Prefrontal Cortex
MRI: magnetic resonance imagery
MSE: multiscale entropy
MTG: middle Temporal Gyrus
PCC: posterior cingulate cortex
PPG: photoplethysmographic
SE: sample entropy
SPL: Superior Parietal Lobule
STG: Superior Temporal Gyrus
TBI: traumatic brain injury
UWS: unresponsive wakefulness syndrome, previously persistent vegetative syndrome (PVS)
WM: white matter.
