# Supplementary material for: A Heartbeat Away From Consciousness: Heart Rate Variability Entropy Can Discriminate Disorders of Consciousness and Is Correlated With Resting-State fMRI Brain Connectivity of the Central Autonomic Network
Source: Front Neurol. 2018 Sep 12;9:769. doi: 10.3389/fneur.2018.00769 (PMC6145008; doi:10.3389/fneur.2018.00769)
Supplement: Supplementary file 1 [file Data_Sheet_1.PDF]

Supplementary Material

A Heartbeat Away From Consciousness: Heart Rate Variability Entropy can discriminate disorders of consciousness and is correlated with resting-state fMRI brain connectivity of the Central Autonomic Network

Francesco Riganello*+, Stephen Karl Larroque*+, Mohamed Ali Bahri, Lizette Heine, Charlotte Martial, Manon Carrière, Vanessa Charland-Verville, Charlène Aubinet, Audrey Vanhaudenhuyse, Camille Chatelle, Steven Laureys, Carol Di Perri

*** Correspondence:** stephen.larroque@uliege.be, f.riganello@istitutosantanna.it

+ These authors contributed equally to this work.

Appendix A: Machine learning models for the complexity index

This section describes the simulation of multiple machine learning approaches to solve the problem of diagnostic classification of MCS and UWS patients, based on the analysis of the Complexity Index in short (CI_s) and long time scale (CI_l) as input features.

Each algorithm was tested with both variables and then with only the CI_l variable (chosen based on the best performance of One-R classifier)

The algorithms tested were based on different approaches, and were chosen based on previous studies comparing the efficiency of various machine learning models (Wahbeh, A. H et al, International Journal of Advanced Computer Science and Applications, 2011):

- Association rule: One-R
- Decision tree learning: J48 (a decision tree is used as predictive model)
- Support vector machines (SVMs): Sequential minimal optimization (SMO). SVM are a set of related supervised learning methods used for classification and regression.
- Regression: Logistic regression, which is a simple linear regression with a *logit* function to bound the result to range [0, 1] in order to allow for classification.
- Bayesian: Naive Bayes.

Highlighted in bold is the best and simplest algorithm for the diagnostic classification of MCS and UWS. However all classifiers gave a high rate of correct classification of MCS and UWS patients.

These simulations were conducted in Weka 3.8 (Frank, E. et al, 2016), programmed in Java.

	Attribute									
Training set	CI_s & CI_l	CI_l	CI_s & CI_l	CI_l	CI_s & CI_l	CI_l	CI_s & CI_l	CI_l	CI_s & CI_l	CI_l
Classifier	One-R		J48		SMO		Naive Bayes		Logistic	
True positive (MCS) rate (%)	94	**94**	94	94	94	88	94	94	88	71
True negative (UWS) rate (%)	93	**93**	93	93	79	93	79	86	86	93
False negative rate (%)	6	**6**	6	6	6	13	6	6	13	13
False positive rate (%)	7	**7**	7	7	21	7	21	14	14	7
Precision MCS classification (%)	94	**94**	94	94	83	94	83	88	88	94
Precision UWS classification (%)	93	**93**	93	93	92	87	92	92	86	87
Accuracy (%)	93	**93**	93	93	87	90	87	90	87	90
F1-score (%)	94	**94**	94	94	88	90	88	91	88	90
Matthews Correlation Coefficient [-1:1]	0.87	**0.87**	0.87	0.87	0.74	0.80	0.74	0.80	0.73	0.80

Table S.1: Training set (the whole S1 group) results for each machine learning algorithm

	Attribute									
10-fold cross validation test set	CI_s & CI_l	CI_l	CI_s & CI_l	CI_l	CI_s & CI_l	CI_l	CI_s & CI_l	CI_l	CI_s & CI_l	CI_l
Classifier	One-R		J48		SMO		Naive Bayes		Logistic	
True positive (MCS) rate (%)	88	**88**	88	94	88	81	81	88	81	81
True negative (UWS) rate (%)	86	**93**	79	86	64	85	79	86	71	85
False negative rate (%)	13	**13**	13	6	13	13	19	13	19	13
False positive rate (%)	14	**7**	21	14	36	7	21	14	29	7
Precision MCS classification (%)	88	**94**	82	88	74	87	81	88	76	87
Precision UWS classification (%)	86	**87**	85	92	82	80	79	86	77	80
Accuracy (%)	87	**90**	83	90	77	83	80	87	77	83
F1-score (%)	88	**90**	85	91	80	84	81	88	79	84
Matthews Correlation Coefficient [-1:1]	0.73	**0.80**	0.67	0.80	0.54	0.67	0.60	0.73	0.53	0.67

Table S.2: 10-fold cross validation test results for each machine learning algorithm

	Attribute									
S2 subgroup set	CI_s & CI_l	CI_l	CI_s & CI_l	CI_l	CI_s & CI_l	CI_l	CI_s & CI_l	CI_l	CI_s & CI_l	CI_l
Classifier	One-R		J48		SMO		Naive Bayes		Logistic	
True positive (MCS) rate (%)	92	**92**	92	92	92	92	92	92	92	92
True negative (UWS) rate (%)	100	**100**	100	100	70	80	60	80	70	80
False negative rate (%)	8	**8**	8	8	8	8	8	8	8	8
False positive rate (%)	0	**0**	0	0	30	20	40	20	30	20
Precision MCS classification (%)	100	**100**	100	100	79	85	73	85	79	85
Precision UWS classification (%)	91	**91**	91	91	88	89	86	89	88	89
Accuracy (%)	95	**95**	95	95	82	86	77	86	77	86
F1-score (%)	96	**96**	96	96	85	88	81	88	85	88
Matthews Correlation Coefficient [-1:1]	0.91	**0.91**	0.91	0.91	0.64	0.73	0.55	0.73	0.64	0.73

Table S.3: S2 subgroup set (i.e., subjects of the fMRI subgroup) results for each machine learning algorithm

Appendix B: Additional information on fMRI analysis

ID	Rejection reason	CRS-R diagnosis
4	Too much movement	UWS
6	Metal artifacts	MCS
8	Bad image quality (e.g., too low contrast)	UWS
13	Metal artifacts, bad image quality	UWS
15	Too much damage	UWS
23	Metal artifacts	MCS
26	Metal artifacts, inhomogenous signal loss	MCS
28	Bad image quality	MCS
29	Bad FOV (missing cortical matter)	MCS

Table S.4: List of rejected subjects from the S1 group for the fMRI analysis.

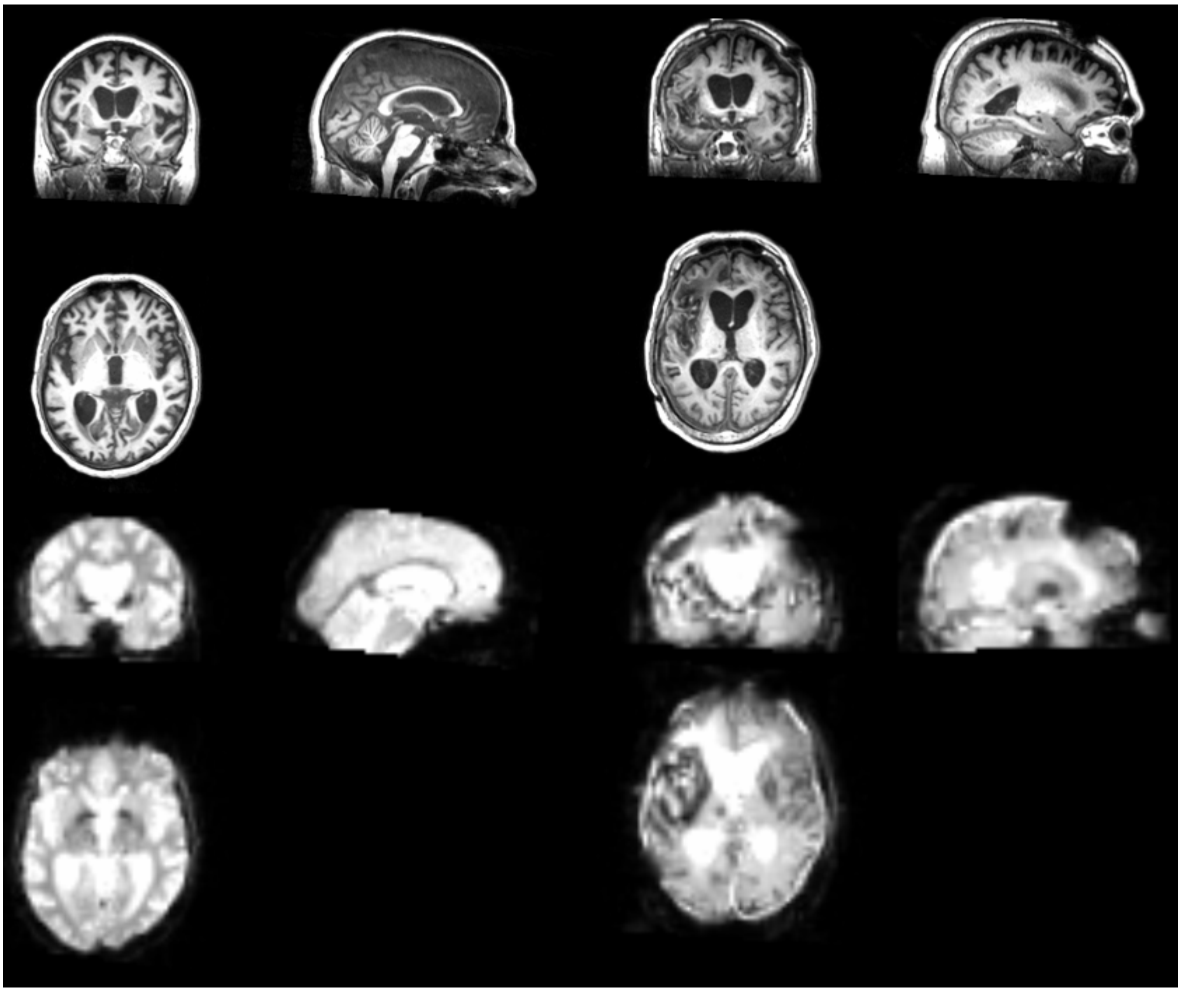

Figure S.1: MRI image examples. On the left, the images from an included subject, with good quality images. On the right, the images from a rejected subject (see Table S.4). The first row show the T1-weighted images, the bottom row the EPI BOLD images. We can see the images from the rejected subject display extensive artifacts such as tissues hyper/hypointensities, metal artifacts and movement artifacts, which would prevent the segmentation of the structural and the analysis of the BOLD timeseries.

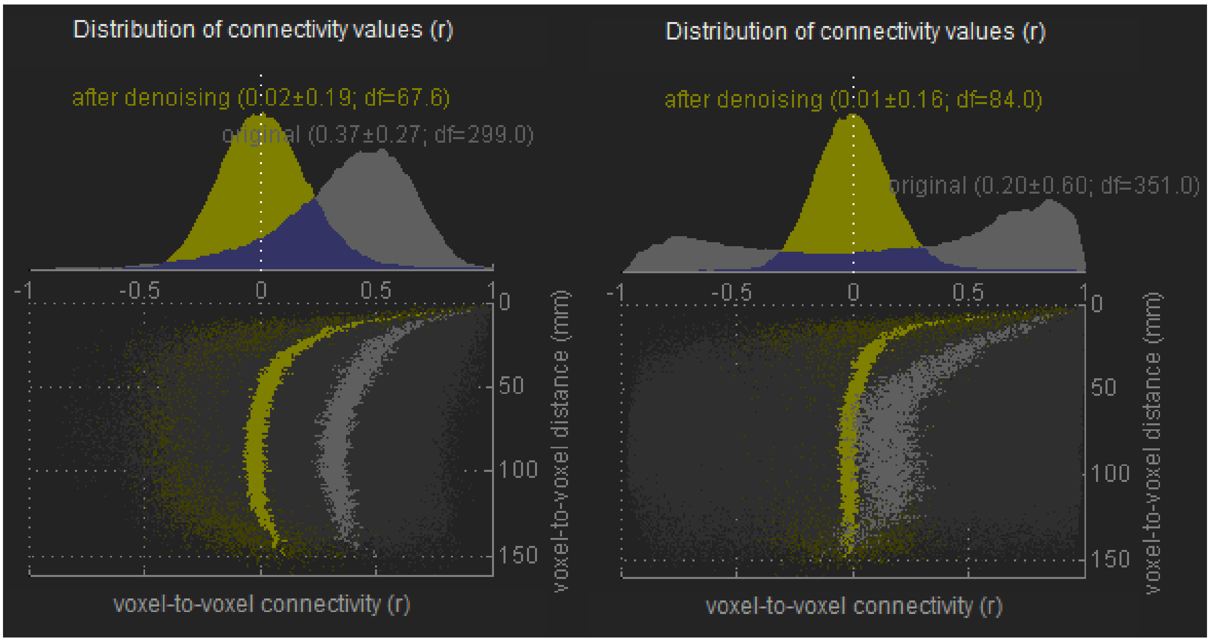

Figure S.2: Examples of BOLD signal shape before (grey/blue) and after (yellow) denoising using aCompCor. On the left, the images from an included subject, with a good signal shape. On the right, the images from a rejected subject (see Table S.4), where the brain images quality was sufficient but with an artifactual BOLD signal.

Seed	Cluster	Size (in voxels)	Mass	Mass p-FWE
ICC	-56 -28 -06	91	432	0.089
Insula	-12 +22 +60	121	493	0.094
Paracingulate	-50 -34 -14	197	904	0.034
	-38 +28 +04	136	535	0.095

Table S.5: Statistical significance table for CI_S. Statistical significance was considered at non-parametric permutation test voxel-wise p-uncorrected < 0.001 and cluster-mass FWE < 0.1. Seeds were chosen following previous literature (1).

Seed	Cluster	Size (in voxels)	Mass	Mass p-FWE
ICC	-36 +32 +30	99	469	0.066
Paracingulate	+48 +56 +00	142	619	0.063
Auditory STG	-30 -50 +60	297	1278	0.01
FP DLPFC	-32 +38 +06	317	1345	0.008
	+48 +46 +10	227	930	0.028

Table S.6: Statistical significance table for CI_L. Statistical significance was considered at non-parametric permutation test voxel-wise p-uncorrected < 0.001 and cluster-mass FWE < 0.1. Seeds were chosen following previous literature (1).

1. Demertzi A, Antonopoulos G, Heine L, Voss HU, Crone JS, de Los Angeles C, Bahri MA, Di Perri C, Vanhaudenhuyse A, Charland-Verville V, et al. Intrinsic functional connectivity differentiates minimally conscious from unresponsive patients. *Brain* (2015) **138**:2619–2631. doi:10.1093/brain/awv169

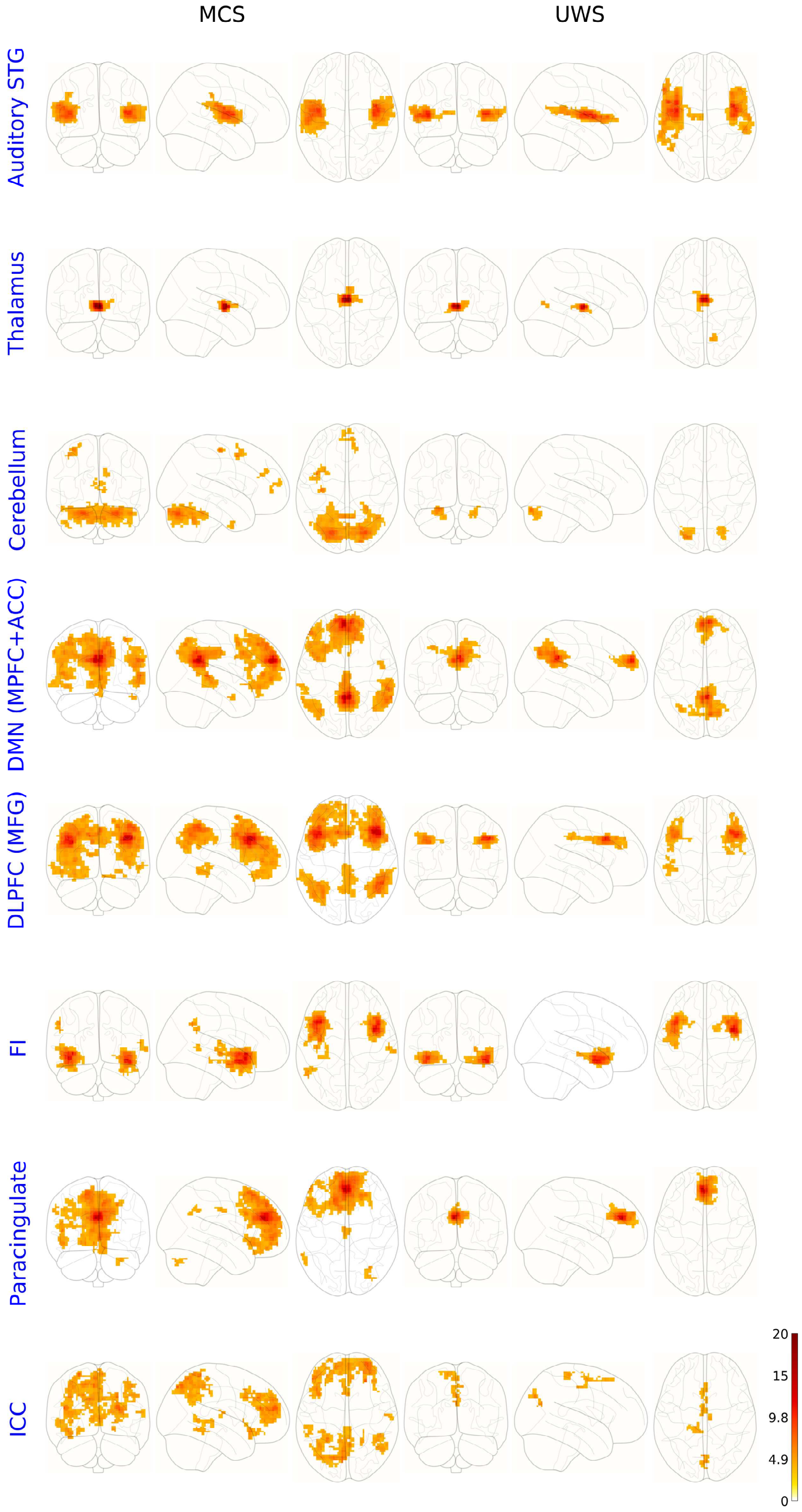

Figure S.3: Average functional MRI connectivity of each group, for each seed and hypothesis-free intrinsic connectivity contrast (ICC) analyses. The complexity index measures were not used here.

Age standardized to unitary variance and centered to the mean was regressed. Statistical significance was considered at non-parametric permutation test voxel-wise p-uncorrected < 0.001 and cluster-mass FDR < 0.1.

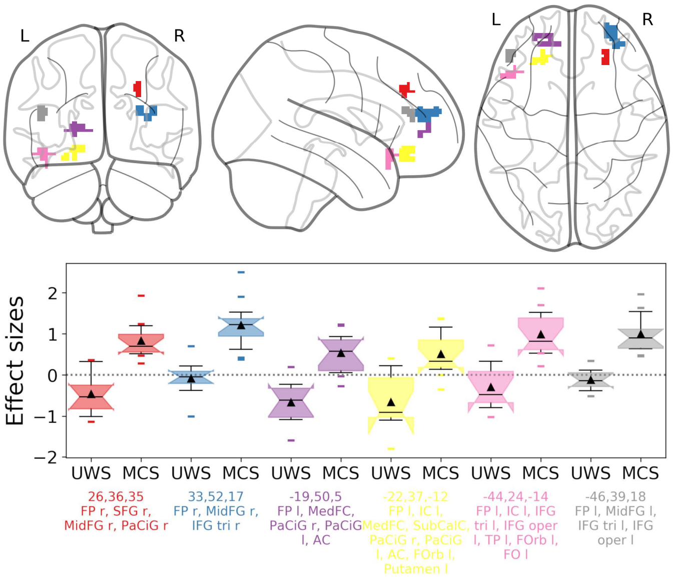

Figure S.4: Result of the difference functional connectivity using the contrast MCS > UWS, with standardized and centered age regressed, for the ICC analysis. None of the seed-based analyses showed statistically significant results for this contrast. The complexity index measures were not used here. Statistical significance was considered at non-parametric permutation test voxel-wise p-uncorrected < 0.001 and cluster-mass FDR < 0.1.

**Seed**	**Cluster**	**Size (in voxels)**	**Mass**	**Mass p-FWE**
ICC	+30 +56 +16	109	458	0.098
(no Complexity	-20 +38 -08	87	360	0.098
Index)	-42 +22 -14	63	269	0.099
	-14 +46 +04	62	244	0.099
	-48 +38 +18	55	226	0.099
	+28 +34 +34	44	214	0.099

Table S.7: Statistical significance table for the ICC analysis on the contrast MCS > UWS without the CI measures. Statistical significance was considered at non-parametric permutation test voxel-wise p-uncorrected < 0.001 and cluster-mass FDR < 0.1.

Appendix C: Additional statistical tests and figures on complexity index

This section describes additional figures on the dataset and statistical tests on various covariates.

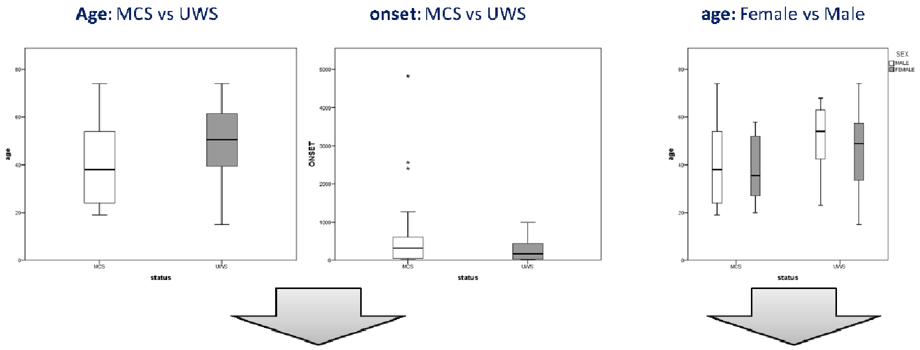

	age	ONSET
U di Mann-Whitney	91,000	80,000
W di Wilcoxon	244,000	171,000
Z	-1,622	-1,482
P exact	,107	,143

status		age	ONSET
MCS	U di Mann-Whitney	33,500	23,000
	W di Wilcoxon	78,500	78,000
	Z	-,241	-1,510
	P exact	,833	,146
UWS	U di Mann-Whitney	26,500	14,500
	W di Wilcoxon	62,500	29,500
	Z	-,578	-,807
	P exact	,592	,456

Figure S.5: Significance tests of the influence of age and onset covariates

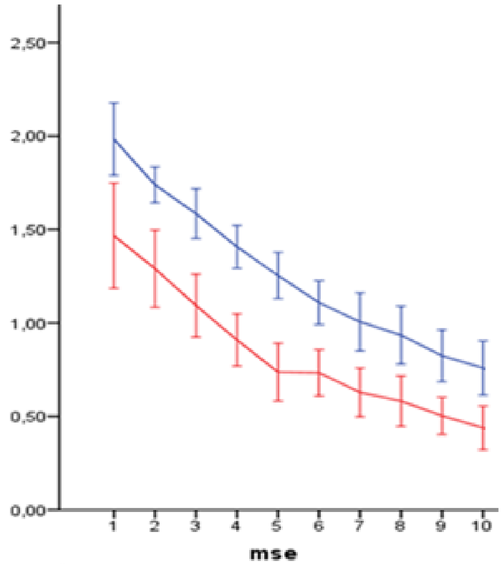

Figure S.6: Mean and standard deviation of the MSE values over time scales 1 to 10, for the MCS group (blue) and UWS group (red)

	Time Domain		Frequency Domain	
	SDNN	RMSSD	LF/HF	nuLF
S1	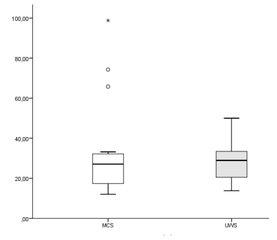	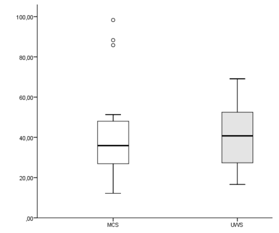	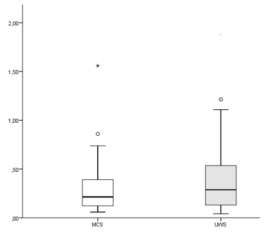	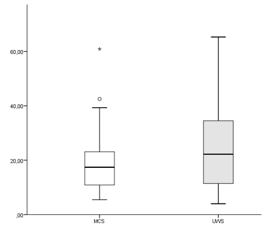
	Z=-1.143; p=.131	Z=-.561; p=.294	Z=-.291; p=.395	Z=-.291; p=.395
S2	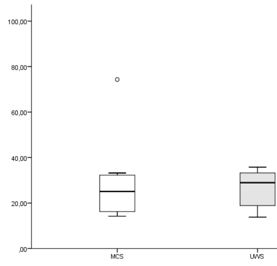	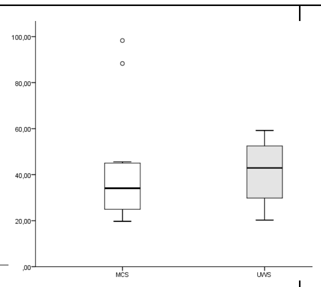	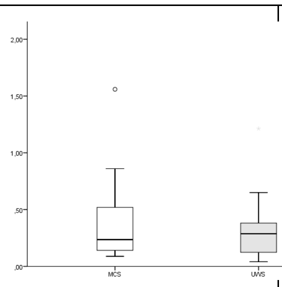	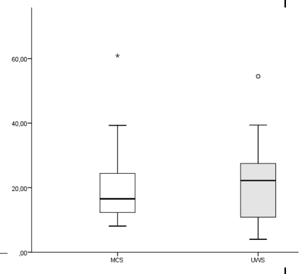
	Z=-.900; p=.197	Z=-.900; p=.208	Z=-1.111; p=.150	Z=-1.111; p=.150

Figure S.7: Time domain (HRV, RMSSD) and Frequency domain (LF/HF, nuLF) statistical analysis comparing UWS and MCS patients summarized as a box plot. The 1st row compares the entire group of patients S1 (n=30), while the 2nd row compares the subgroup of patients S2 (n=21) who underwent fMRI analysis. The boxes range from Q1 to Q3, while the whiskers are defined at the 1.5 interquartile range, and the black lines are the medians, points are outliers. Below each boxplot are the Mann-Whitney's test results of MCS vs UWS comparison.

Time Frequency

SDNN is the standard deviation of the peak-to-peak intervals and represent all the cyclic components responsible for the variability in the period of recording considerated. RMSSD is calculated as the square root of the mean of the squares of the successive differences between adjacent RR peaks. This parameter is highly correlated with the parasympathetic nervous system.

Frequency domain

Frequency domain analysis is performed by fast Fourier transform (FFT) and the Power Spectrum Density is calculated for specific frequency bands. In particular the low frequency (LF) band (0.04-0.15 Hz) is modulated mainly by sympathetic nervous system while the high frequency(HF) band (0.15-0.50 Hz) is driven almost exclusively by parasympathetic nervous system. The ratio of LF to HF power is often used as a metric of sympathetic—parasympathetic balance, while the nuLF is the normalized value of LF, calculated as LF/(LF+HF).

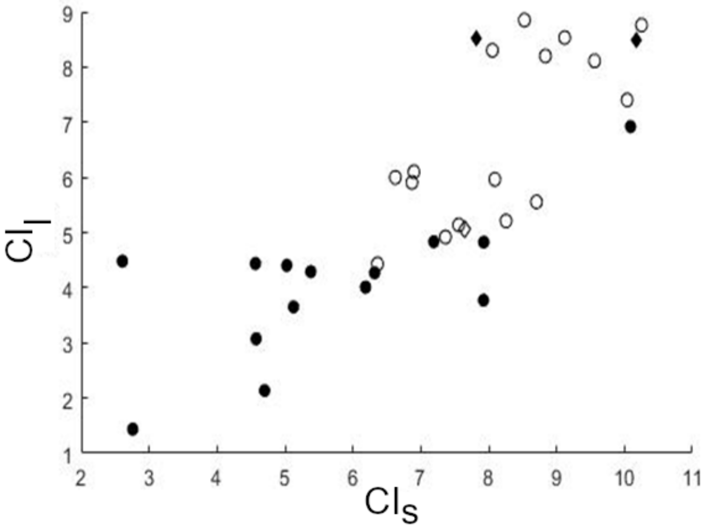

Figure S.8: Dispersion graph of CI_l and CI_s for the S1 group with rejected non-sedated patients as diamonds.
